# A Canine Case of *Nocardia africana* Infection Detected by Matrix-Assisted Laser Desorption Ionization—Time-of-Flight Mass Spectrometry

**DOI:** 10.3390/vetsci9060265

**Published:** 2022-06-01

**Authors:** Ji-Seon Yoon, Hyungjae So, Beomsung Joo, Jihong Park, In-Seong Jeong, Gi-Jong Lee, Jinho Park

**Affiliations:** 1 Department of Veterinary Internal Medicine, Konkuk Veterinary Medical Teaching Hospital, Seoul 05029, Korea; jiseonyoon@konkuk.ac.kr; 2 Royal Animal Medical Center, Seoul 02117, Korea; broscow@hanmail.net (H.S.); tiger0606@hanmail.net (B.J.); padak2000@naver.com (J.P.); jung45457@hanmail.net (I.-S.J.); gang8713@hanmail.net (G.-J.L.); 3 Department of Veterinary Internal Medicine, College of Veterinary Medicine, Jeonbuk National University, Iksan 54596, Korea

**Keywords:** dog, skin, nocardiosis, *Nocardia africana*, MALDI-TOF analysis

## Abstract

Nocardiosis, a rare infectious disease in dogs and cats, is caused by Gram-positive aerobic actinomycetes of the genus *Nocardia*. A one-year-old castrated male Great Dane was presented with clinical signs of an ulcerated nodule on the right ear, which was observed after two weeks of treatment with cyclosporine and prednisolone due to idiopathic hepatitis. Cytological examination revealed pyogranulomatous inflammatory cells and blanched filamentous rods. To detect infectious agents, serosanguinous discharge of the nodule was subjected to bacterial and fungal cultures. For phenotyping of the infectious agents, colonies on blood agar culture plates were further analyzed by matrix-assisted laser desorption ionization (MALDI)-time-of-flight (TOF) mass spectrometry (VITEK MS). The MALDI-TOF spectra were identified as *N. africana*. Thus, the present case was diagnosed as cutaneous nocardiosis. The skin lesions of ulcerated nodules with fistulous tracts were gradually resolved by the administration of meropenem (8 mg/kg TID, IV) and doxycycline (5 mg/kg BID, PO). Although complete resolution of the skin lesions was observed on day 91 after the initial presentation, single administration of doxycycline was continued until day 198 after the initial presentation to prevent recurrence. To the best of our knowledge, this is the first report of *Nocardia africana* infection in a dog. In addition, our results show that MALDI-TOF mass spectrometry analysis could be a useful tool for the detection of *Nocardia*. spps.

## 1. Introduction

Nocardiosis is a rare infectious disease in dogs and cats that is caused by Gram-positive, nonmotile, aerobic actinomycetes in the genus *Nocardia*. *Nocardia* spp. are common in organic materials, soil, water, and plants, and usually induce pyogranulomatous inflammation in immunocompromised humans and animals [1,2,3]. Accordingly, inhalation or subcutaneous transmission of *Nocardia* spp. induces pyogranulomatous to suppurative inflammatory reactions either in localized organs (skin or lungs), or disseminates to more than one organ. Therefore, it can be classified into three clinical presentations, cutaneous, pulmonary, and systemic forms [1,2,3]. Among these three presentations, cutaneous nocardiosis is the most common and manifests as mycetoma, regional lymphadenitis, ulceration, and abscesses with fistulous tracts that drained a serosanguineous discharge. Lesions are usually observed on the extremities, facial area, and neck [4]. In animals, the majority of reported cases are caused by species of the *Nocardia (N.) asteroides* complex. Other common pathogenic species include *N. brasiliensis*, *N. nova* and *N. otitidiscaviarum* [5,6]. *N. africana*, *N. elegans*, and *N. tenerifensis* have rarely been reported in cats and *N. abscessus* has rarely been reported in dogs [5,6,7,8]. However, to the best of our knowledge, there have been no reports of *N. africana* infection in dogs.

The routine diagnosis of nocardiosis in veterinary medicine is based on histopathological analysis and microbiological culture [5]. Molecular methods, such as 16S rRNA, heat shock protein and essential secretory protein–based polymerase chain reaction (PCR), have been used to confirm the phenotypic diagnosis of *Nocardia* spp. [6]. Recently, mass spectrometry-based proteomic analysis, including matrix-assisted laser desorption ionization (MALDI)-time-of-flight (TOF), has emerged as a rapid and reliable methodology that allows the phenotypic diagnosis of a great variety of microorganisms of clinical interest [9]. Routine extraction methods for detection of *Nocardia* spp. by MALDI-TOF analysis can be used to reliably identify *Nocardia* spp. [10,11].

## 2. Case Description

**Figure 1:**
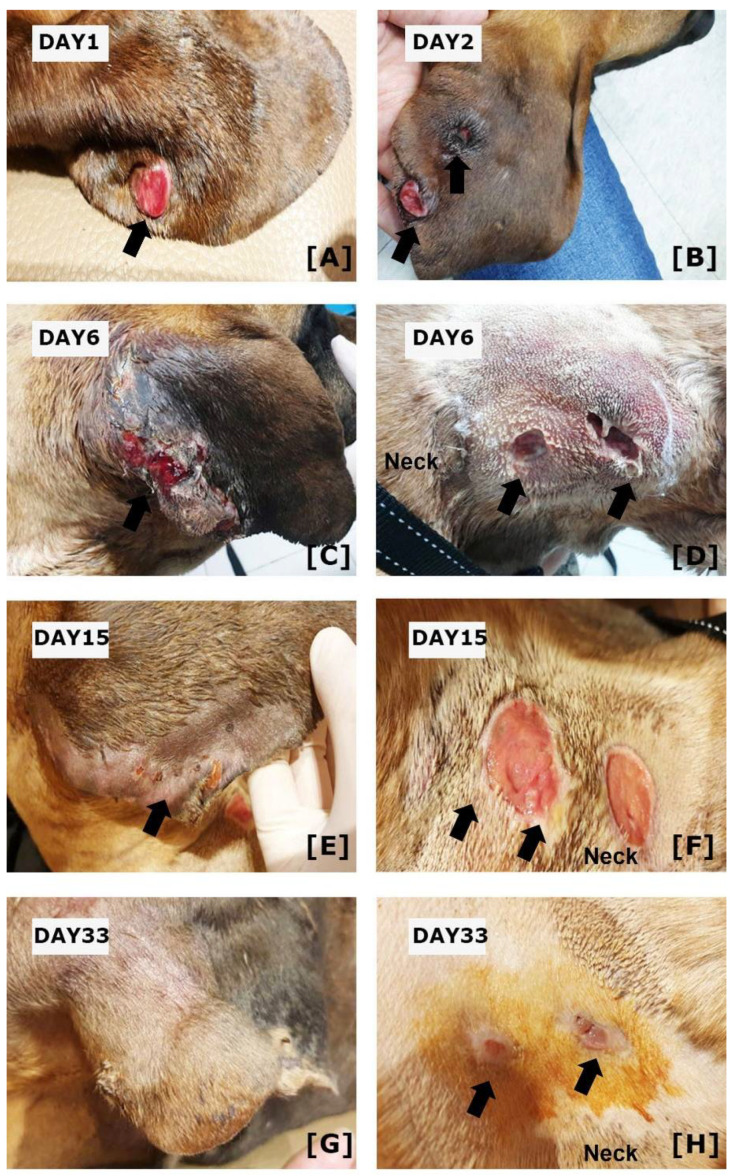
Clinical Features of the Present Case. (**A**) At the initial presentation, ulcerated nodules on the right ear were seen. (**B**) On day two, another lesion with ulceration was observed on the right pinna. (**C**) On day 6, the ulcerative lesions on right pinna have enlarged, (**D**) and ulcerations with fistulous tract were observed on the neck. (**E**) After administration of meropenem and doxycycline, the ulcerations on right pinna have gradually resolved on day 15. (**F**) However, the ulcerations on the neck were still observed. (**G**) On day 33, the skin lesions on the right pinna were resolved and show hair regeneration. (**H**) Ulcerative lesions on the neck also show skin regeneration. Arrows indicate skin lesions.

A one-year-old castrated male Great Dane living outdoors was presented with history of nodules on his right ear for one week. The dog had a prior medical history that included recent diagnosis with idiopathic hepatitis, for which he had been receiving cyclosporine (10 mg/kg SID, PO, Neoral; Novartis, Switzerland) and prednisone (1 mg/kg BID, PO, Sorondo; Yuhan Co. Ltd., Seoul, Korea). The nodules were first observed after about two weeks of immunosuppressive therapy. At the initial presentation, a single button-like nodule was observed on the right pinna (Figure 1A). The nodule was firm, and the entire surface was ulcerated. The next day, the lesions became swollen, and another ulcer with a fistulous tract appeared on the right pinna (Figure 1B). The fistulous tract drained the serosanguinous discharge. On cytological examination of the ulcerated lesions, the main inflammatory cells were degenerative neutrophils, macrophages, and lymphocytes. In addition, branched filamentous rods were observed (data not shown). Based on the cytological findings, deep bacterial infection, deep fungal infection, foreign body reaction and sterile pyogranuloma/granuloma syndrome were included as differential diagnosis. To investigate systemic condition of the patient, we performed complete blood count and serum chemistry that revealed neutrophilia (38.88; reference range: 6–17 K/uL), hypoalbuminemia (2.1; reference range: 2.3–3.9 g/dL), increased ALT(122; reference range: 3–50 U/L), increased AST (63; reference range: 10–37 U/L), increased total bilirubin (2.6; reference range: 0.1–0.7 mg), increased amylase (1156; reference ranges: 388–1007 U/L), decreased total calcium (8.8; reference range: 9.1–11.7 mg). Hypoalbuminemia and increased liver enzymes and increased total bilirubin was thought be associated chronic hepatitis of the patients. The serosanguinous discharge of ulcerated lesions was collected by swabbing fistulous tract on ulcerative lesions and subjected to fungal and bacterial cultures and PCR for detection of mycobacteria (POPANILAB, Gyeonggi, Korea). Cefazolin (20 mg/kg TID, IV, Cefazolin injection; Yuhan Co. Ltd., Seoul, Korea) and enrofloxacin (10 mg/kg, SID, IV, Baytril; Bayer AG, Barmen, Germany) were administered during the waiting period.

The culture colonies of serosanguinous discharge of ulcerated lesions were further investigated by MALDI-TOF mass spectrometry. The intensity of the specimen showed 99% of confidence value with *N. africana.*

**Figure 2:**
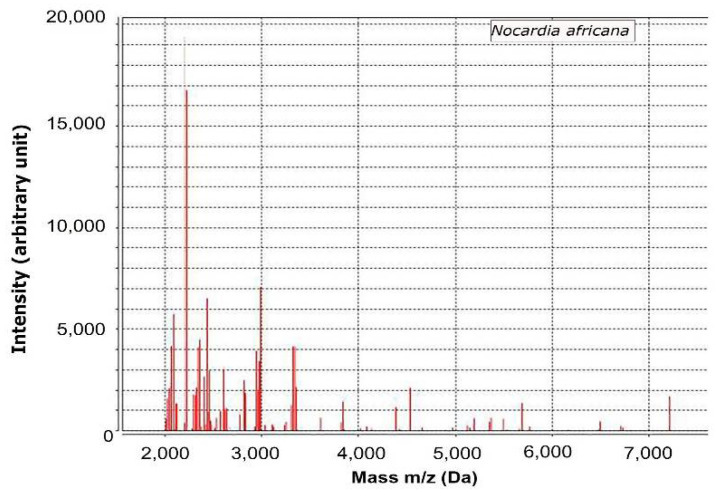
Analysis of MALDI-TOF mass spectrometry in the present case.

The specimens were cultured on blood agar plates and incubated at 37 °C. On day two of incubation, chalky white and cotton appearance colonies were observed on blood agar plates. The colonites were further subjected to MALDI-TOF mass spectrometry (VITEK MS v.3 bioMérieux, Inc., Durham, NC, USA) for the phenotyping of infectious agents. Briefly, colonies were picked with a sterile loop and then prepared for analysis using a Mycobacterium/Nocardia reagent kit (Vitek MS; bioMérieux, Inc., Durham, NC, USA) according to the manufacturer’s instructions. Mechanical disruption using 0.5 mm-diameter glass beads and bead beating for 5 min were performed, and then the samples was incubated with 70% ethanol for 10-min at room temperature and then protein was extracted using 70% formic acid. One microliter of the protein extract was then applied to a single spot on the MALDI target plate (Vitek MS-DS; bioMérieux, Inc., Durham, NC, USA), allowed to dry, and 1 μL of alpha-cyano-4-hydroxycinnamic acid (Vitek MS-CHCA matrix; bioMérieux, Inc., Durham, NC, USA) was added. After the plate was dried, spectra were acquired using MALDI-TOF mass spectrometry (VITEK MS v.3 bioMérieux, Inc., Durham, NC, USA) and compared with the VITEK MS database, which includes more than 15,000 distinct microorganisms, including *Nocardia* spp. The spectra of the specimen were identified as *N. africana* (Figure 2) showing a 99% of confidence value. Thus, the present case was diagnosed as nocardiosis. In contrast, no infectious agents were detected by fungal culture or PCR to detect mycobacteria (data not shown). Further antibiotic sensitivity tests were performed using automated minimum inhibitory concentration testing (VITEK 2 AST, bioMérieux, Inc., Durham, NC, USA). While waiting for the results of antibiotic sensitivity tests, they were changed to meropenem (8 mg/kg TID, IV, MEROPENEM Daewoong; Daewoong Pharmaceutical, Seoul, Korea) and trimethoprim-sulfamethoxazole (21 mg/kg BID, PO, Septrin; Samil Pharmaceutical Co., Seoul, Korea) on day four, as meropenem in addition to trimethoprim-sulfonamide are known to be effective in nocardiosis. However, the skin lesions did not resolve, and another ulcerative lesion with a fistulous tract appeared on the neck 6 days after initial presentation (Figure 1C,D). Antibiotic sensitivity tests revealed that *N. africana* isolated from this dog were resistant to trimethoprim-sulfonamides, and the antibiotics were changed to meropenem and minocycline (5 mg/kg BID, PO, Minocin; SK chemical, Gyeonggi-do, Korea)) on day nine. The results of antibiotic sensitivity tests are shown in Table 1. Although ulceration and serosanguinous discharge gradually resolved after treatment with meropenem and minocycline, the antibiotics were changed to meropenem and doxycycline (5 m/kg BID, PO, Doxycycline tab; Youngpoong pharmaceuticals, Korea) on day 14. On day 15, the ulceration of the right ear was almost resolved, and also indicated by regrowth of hair (Figure 1E). Ulcerations of the neck were still present (Figure 1F). On day 33, complete resolution of the skin lesion on the right ear was observed (Figure 1G), and skin regeneration was observed in the neck (Figure 1H). On day 45, the administration of meropenem was discontinued and single administration of doxycycline was used. On day 91, complete resolution of the skin lesions in the right ear and neck was observed. Administration of doxycycline was continued until day 198 to prevent the recurrence of clinical signs. No relapse was observed as of 12 months after completion of antibiotic therapy, at which time the patient was lost to follow-up.

## 3. Discussion

In this report, we describe the first case report confirming cutaneous nocardiosis caused by *N. africana* in the dog. The present case showed ulcerated nodules and a fistulous tract with serosanguinous discharge after two weeks of immunosuppressive drug administration. In this report, we performed MALDI-TOF spectra analysis using VITEK MS instrument revealed that the culture colonies of the skin specimen were *N. africana*. The skin lesions gradually resolved with administration of meropenem and doxycycline. Therefore, the present case was diagnosed as cutaneous nocardiosis caused by *N. africana* infection.

*N. africana*, which was first identified in human patients with chronic lung disease, has rarely been reported in humans [12]. In veterinary medicine, two cases of *N. africana* have been reported in cats [7,8]. It was first identified in cats with mycetoma, which showed white-yellow nodules with a fistulous tract on the tail and abdomen. The nucleotide sequence of the 16S ribosomal DNA of the clinical isolate corresponded to that of a reference strain of *N. africana* [7]. Another report of feline nocardiosis caused by *N. africana* described fever, submandibular lymphadenitis, and an ulcerated mass with serosanguineous discharge in the left mandible. Radiography of the mandible also revealed osteomyelitis with intense bone proliferation and osteolysis. Sequencing analysis of the 16S rDNA identified the organism as *N. africana* based on 99% sequence similarity with the reference sequence [8]. In this case of *N. africana* infection in a dog, the skin lesions of ulcerated nodules with a fistulous tract were similar to those in previous reports of *N. africana* infections in cats.

As nocardiosis can induce fatal systemic infections, rapid diagnosis and appropriate treatment are required. Furthermore, differentiating *Nocardia* spp. at the species level provides more information on the predicted response to antibiotic therapy and patient prognosis [13]. Recently, mass spectrometry-based proteomic analysis, including MALDI-TOF, has been reported to provide rapid and reliable diagnosis of disease including canine nocardiosis [14,15]. *Nocardia* spp. detected by MALDI-TOF showed high correspondence at the species level with those detected by PCR targeting 16S ribosomal DNA [10,11]. The time to perform MALDI-TOF analysis was shorter than that of classical molecular testing [10,11]. In this case, MALDI-TOF analysis using VITEK MS instrument was conducted within 1 day, after observing colonies on plates. Similarly, a previous report [14] of canine nocardiosis caused by *N. veterana* successful treatment could be achieved by early diagnosis of nocardiosis by MALDI-TOF analysis. Therefore, MALDI-TOF analysis might be useful for rapid diagnosis nocardiosis and identification of the specific levels of *Nocardia*.

Treatment of nocardiosis usually requires long-term antibiotic administration. Trimethoprim-sulfamethoxazole has long been used as first-line antibiotic therapy for nocardiosis, but resistance to this treatment has recently emerged [16]. In two previous reports of feline nocardiosis caused by *N. africana*, clinical signs were not controlled by treatment with trimethoprim-sulfamethoxazole [7,8]. Similarly, *N. africana* isolated from this dog were resistant to trimethoprim-sulfonamides, and administration of trimethoprim-sulfamethoxazole did not resolve the skin lesions. Other first-choice drugs for the treatment of nocardiosis include amoxicillin-clavulanate, imipenem, and some cephalosporins. It has also been reported that clinical signs can improve by combining other drugs such as ampicillin, linezolid, doxycycline, erythromycin, and minocycline [13]. The present case also used a combination therapy of meropenem and doxycycline, and complete resolution of skin lesions was observed with these treatments. Furthermore, even after clinical signs have resolved, long-term antibiotic administration is required due to the fact that clinical relapses can be observed after short-term protocols [1,2]. In the present case, even though clinical signs already resolved on day 91, antibiotic therapy was continued until day 198, and no relapse of nocardiosis was observed as of 12 months after stopping treatments.

*Nocardia* infection is more likely in immunosuppressed individuals, as previously reported [1,2]. In the present case, *Nocardia* infection was also observed in an immunocompromised dog treated with prednisolone and cyclosporine. *Nocardia* infection should be considered when cutaneous nodules and ulcers are observed in dogs during ongoing administration of immunosuppressive drugs, and culture and phenotyping of infectious agents need to be performed.

One of the potential limitations of this case report is that other diagnostic tests such as histopathological analysis were not performed. In addition, other tests to investigate systemic condition such as x-ray, ultrasonography, and CT scan were not performed and thus concurrent systemic signs of nocardiosis were not completely ruled out. Further tests such as histopathological analysis and CT scan would provide further information to diagnose cutaneous nocardiosis in this case.

## 4. Conclusions

In this report, we describe diagnosis of cutaneous nocardiosis caused by *N. africana* infection in a dog using MALDI-TOF of VITEK MS instrument. To the best of our knowledge, this is the first report of an *N. africana* infection in a dog. MALDI-TOF mass spectrometry was a useful tool for the detection of *Nocardia* spp.

## Figures and Tables

**Table 1:** Antibiotic sensitivity results of *Nocardia africana* in this case.

Antibiotics	Sensitivity	Antibiotics	Sensitivity
Amikacin	S	Cefalothin	R
Cefazolin	S	Cefotaxime	R
Cefotetan	S	Cefoxitin	R
Gentamicin	S	Ciprofloxacin	R
Imipenem	S	Doxycycline	R
Kanamycin	S	Penicillin	R
Minocycline	S	Sulphamethox/Trimethoprim	R
Amoxycillin/Clavulanic acid	R	Vancomycin	R
Ampicillin	R		

## Data Availability

The data presented in this study are available upon request from the corresponding author.
